# *VARS2* deficiency drives seronegative myasthenia gravis pathology via mitochondrial, immune, and neuromuscular dysregulation

**DOI:** 10.3389/fimmu.2026.1861435

**Published:** 2026-09-18

**Authors:** Zhaojie Lyu, Bo Yang, Yaobin Zhou, Meng Shi, Zhihao Wang, Sa Song, Xinwen Zhang, Jing Tian

**Affiliations:** 1 Center for Automated and Innovative Drug Discovery, College of Life Sciences, Northwest University, Xi’an, Shaanxi, China; 2 Key Laboratory of Resource Biology and Biotechnology in Western China, Ministry of Education, College of Medicine, Northwest University, Xi’an, Shaanxi, China; 3 Center of Medical Genetics, Xi’an People’s Hospital (Xi’an Fourth Hospital), Xi’an, Shaanxi, China

**Keywords:** immunometabolism, mitochondrial dysfunction, myasthenia gravis (MG), neuromuscular junction, seronegative myasthenia gravis (SNMG), *VARS2*, zebrafish model

## Abstract

Seronegative myasthenia gravis (SNMG) is a clinically heterogeneous subtype of myasthenia gravis (MG) that lacks the classical pathogenic autoantibodies used in routine diagnosis, leaving the underlying mechanisms poorly defined. Integrative genetic and single-cell transcriptomic analyses identified *VARS2* as an MG-associated candidate gene with particular relevance to SNMG. *VARS2* dysregulation was predominantly observed in CD4^+^ T cells, especially in the SNMG subgroup. To evaluate its *in vivo* relevance to SNMG, we established a zebrafish *vars2* knockdown model. *vars2* deficiency induced a myasthenia-like phenotype characterized by impaired locomotor activity, abnormal skeletal muscle development, defective motor neuron formation, and reduced expression of neuromuscular junction-related genes. Furthermore, the loss of *vars2* disrupted mitochondrial homeostasis, evidenced by mitochondrial structural damage, membrane potential loss, increased reactive oxygen species accumulation, and suppression of metabolic pathways. These changes were accompanied by immune dysregulation, marked by CD4^+^ T cell/Th17-associated inflammatory activation, reduced expression of Treg-related genes, and increased neutrophil and macrophage recruitment. Crucially, pharmacological interventions partially reversed these defects through mitochondrial support (CoQ10), acetylcholinesterase inhibition (Neostigmine), and targeted immune suppression (ML604440 for Th17 differentiation, GSK805 for Th17 function, and UNBS5162 for CXCL-related signaling). Together, these findings suggest that VARS2 links mitochondrial dysfunction to immune imbalance and neuromuscular injury in SNMG. Our study highlights mitochondrial immunometabolism as a candidate mechanistic axis and identifies VARS2 as a potential biomarker for SNMG-related immune remodeling.

## 1 Introduction

Myasthenia gravis (MG) is an acquired autoimmune disorder characterized by impairment of neuromuscular junction (NMJ) transmission (1, 2). Based on the spectrum of pathogenic autoantibodies, MG comprises immunologically distinct subtypes (1–3). While most patients harbor autoantibodies against the acetylcholine receptor (AChR) or muscle-specific kinase (MuSK), a subset lacks these classical serological markers and is classified as having seronegative myasthenia gravis (SNMG) (3, 4). Notably, up to 15% of patients with generalized MG and 50% of those with ocular MG are negative for AChR and MuSK antibodies on routine testing (4). A critical clinical challenge in managing SNMG is the absence of definitive serological evidence, which is compounded by profound clinical heterogeneity. This diagnostic ambiguity is particularly evident in early-stage disease or ocular involvement, where SNMG is frequently misdiagnosed as mitochondrial myopathy or other neuromuscular disorders (3, 4). Consequently, there is an urgent need to elucidate the molecular basis of SNMG and to identify mechanism-driven biomarkers for precise stratification and targeted intervention.

Beyond humoral autoimmunity, CD4^+^ T cell-mediated cellular immune dysregulation is a pivotal driver of MG immunopathology (5). CD4^+^ T cells not only participate in B-cell activation and antibody production, but also influence disease progression by secreting inflammatory cytokines and regulating the immune microenvironment (5). Studies have shown that MG patients typically exhibit enhanced Th17 cell-associated inflammatory responses, together with reduced numbers or impaired function of regulatory T cells (Treg cells). The imbalance between Th17 and Treg cells is considered an important mechanism for immune tolerance breakdown and chronic inflammation maintenance (5–7). In some patients with SNMG, enhanced CD4^+^ T cell activation and abnormal Th17/Tfh-related cytokine profiles have also been observed, suggesting that despite the absence of classical autoantibodies, SNMG may still be characterized by a distinct basis of cellular immune imbalance (8). Abnormal immunometabolism may further contribute to immune dysregulation in autoimmune diseases, with mitochondria serving as central regulators of energy metabolism, redox homeostasis, cellular stress, and immune responses (9, 10). In CD4^+^ T cells, mitochondrial dysfunction may promote pro-inflammatory immune responses and weaken immunosuppressive function, thereby driving the initiation and persistence of autoimmune responses (9–11). Concurrently, the neuromuscular system, as a highly energy-demanding tissue, is particularly vulnerable to mitochondrial defects, and impaired mitochondrial translation or oxidative phosphorylation (OXPHOS) can directly lead to neuromuscular injury (9–11). Therefore, mitochondrial dysfunction may represent an upstream mechanism linking immune imbalance and neuromuscular damage in SNMG, although the precise molecular drivers remain unclear.

The mitochondrial valyl-tRNA synthetase, encoded by the *VARS2* gene, is an essential component of the mitochondrial translation machinery. It catalyzes the attachment of valine to mitochondrial tRNA^Val^, thereby maintaining the synthesis of mitochondrially encoded proteins and the function of respiratory chain complexes (12, 13). Mutations or dysregulation of *VARS2* have been linked to mitochondrial translation defects, OXPHOS deficiency, disrupted fatty acid oxidation, and severe multisystem mitochondrial disorders (13–15). Furthermore, members of the aminoacyl-tRNA synthetase family possess noncanonical functions extending beyond protein synthesis, including the regulation of inflammatory and immune responses (16, 17). Given these dual roles, we hypothesized that *VARS2* may act as a critical molecule linking mitochondrial dysfunction, immunometabolic imbalance, and downstream tissue injury (12, 16). However, the spatial expression pattern of *VARS2* in SNMG and its specific contribution to CD4^+^ T cell dysfunction and subsequent NMJ impairment remain unexplored.

In this study, by integrating genome-wide association study (GWAS) data, summary-data-based Mendelian randomization (SMR), and colocalization analyses, we identified *VARS2* as a genetically supported candidate driver of MG. Single-cell transcriptomic profiling further revealed that *VARS2* dysregulation was primarily localized to CD4^+^ T cells, particularly within the SNMG patient subgroup. Utilizing an *in vivo* zebrafish model, we systematically demonstrated that vars2 deficiency precipitates an SNMG-like phenotype, disrupting mitochondrial homeostasis, driving immune-related abnormalities, and impairing neuromuscular development. Our findings unveil the pathogenic contribution of *VARS2* to SNMG and provide novel mechanistic insights into the interplay between immunometabolism and NMJ integrity.

## 2 Results

### 2.1 Integrative Mendelian randomization, colocalization and single-cell transcriptomic analyses identify *VARS2* as a MG-associated candidate gene

To identify candidate causal genes for MG, we performed a SMR analysis using whole-blood *cis*-expression quantitative trait locus (*cis*-eQTL) data from the eQTLGen. Information on the included GWAS, eQTL, and single-cell datasets is summarized in **Supplementary Table 1**. Four genes, *VARS2*, *HLA-DRB5*, *PGAP3* and *CTSH*, were significantly associated with MG (**Figure 1A**; **Supplementary Table 2**), among which VARS2 showed the strongest association with MG. Forest plot analysis showed that higher expression of *HLA-DRB5* and *CTSH* was associated with increased MG risk, whereas lower expression of *VARS2* and *PGAP3* was associated with increased MG risk (**Figure 1B**). Further colocalization analysis showed that only *VARS2* demonstrated strong colocalization evidence with MG risk in whole blood (PP_H4_ = 0.996, *P* < 0.001, **Figure 1C**).

**Figure 1:**
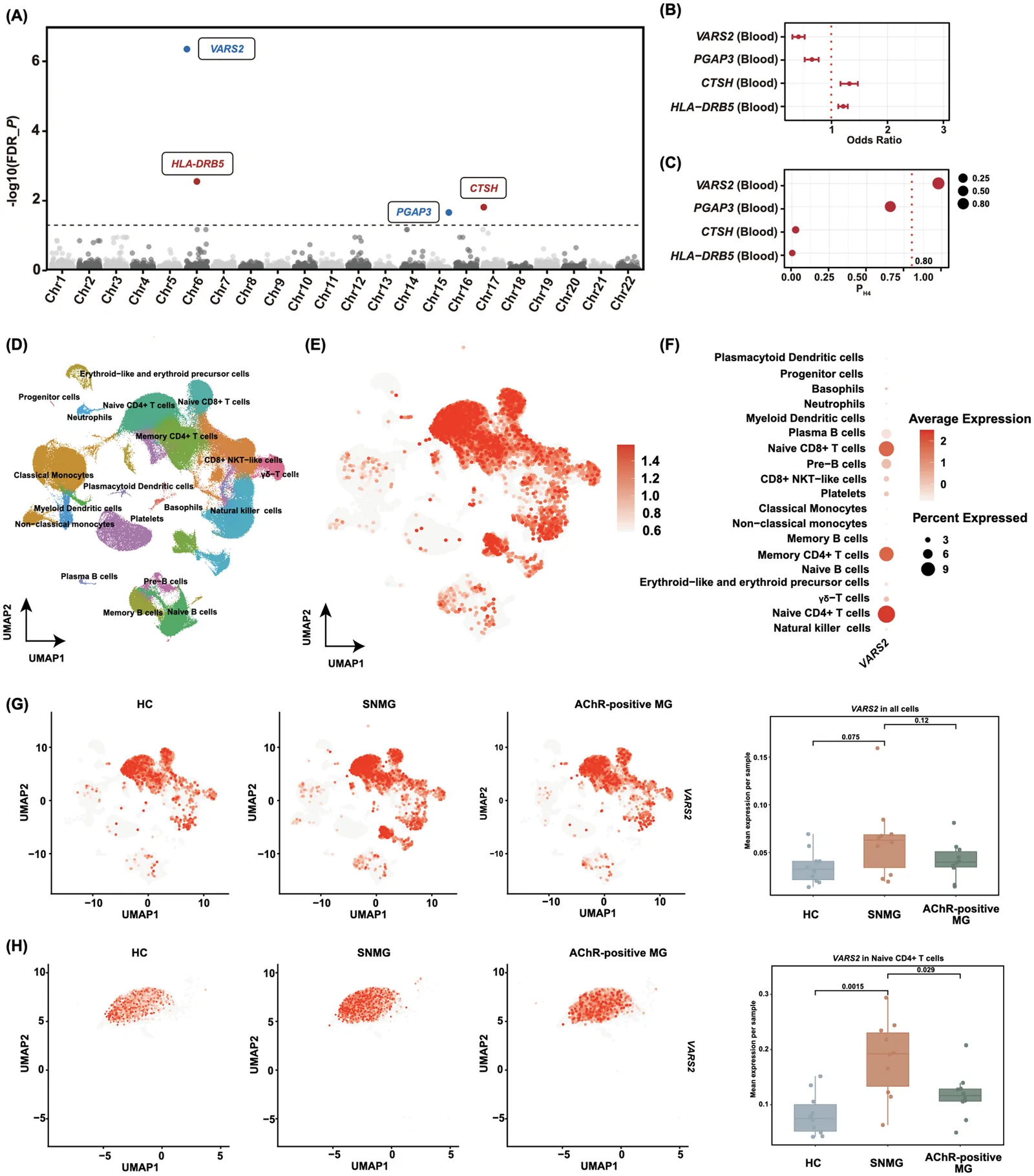
Integrative genetic and single-cell transcriptomic analyses identify *VARS2* as an MG-associated candidate gene. **(A–C)** Genetic analyses of MG using whole-blood cis-eQTL data and GWAS summary statistics. **(A)** SMR analysis showing genes associated with MG risk. **(B)** Forest plot showing the effect directions of the prioritized genes. **(C)** Colocalization analysis of the prioritized genes in whole blood. **(D–H)** Single-cell transcriptomic analysis of peripheral immune cells from healthy controls (HC), seronegative myasthenia gravis (SNMG), and AChR-positive MG patients. **(D)** UMAP plot showing annotated immune cell populations. **(E)** Feature plot showing *VARS2* expression across all immune cells. **(F)** Dot plot showing the average expression and percent expressed of *VARS2* across immune cell subsets. **(G)** Feature plots and quantification of *VARS2* expression across all cells in the HC, SNMG, and AChR-positive MG groups. **(H)** Feature plots and quantification of *VARS2* expression in naive CD4^+^ T cells in the HC, SNMG, and AChR-positive MG groups.

Given the genetic implication of *VARS2* in whole blood data, we next profiled its cellular origin and expression dynamics in the MG immune microenvironment by single-cell transcriptomics of peripheral immune cells from healthy controls (HC), SNMG, and AChR-positive MG patients. Unsupervised clustering and cell-type annotation identified diverse immune populations across cohorts (**Figure 1D**). Uniform Manifold Approximation and Projection (UMAP) showed heterogeneous *VARS2* expression across cell types (**Figure 1E**), with the highest average level in naïve CD4^+^ T cells (**Figure 1F**).

To delineate the disease- and subtype-specific expression pattern of *VARS2*, we compared its transcript abundance across the HC, SNMG, and AChR-positive MG groups. *VARS2* dysregulation was most evident in naïve CD4^+^ T cells, where expression was significantly higher in SNMG than in HC or AChR-positive MG (**Figures 1G, H**; **Supplementary Table 3**). These results identify *VARS2* as a high-confidence MG-associated risk gene and localize its most prominent transcriptional alteration to naïve CD4^+^ T cells in SNMG.

### 2.2 *vars2* depletion compromises skeletal muscle myogenesis and spinal motor neuron development in zebrafish

Having identified *VARS2* as a genetically prioritized candidate gene, we next examined its function *in vivo* using a zebrafish model. VARS2 is highly conserved across vertebrates and exhibits a similar predicted overall structure in humans, mice, and zebrafish (**Supplementary Figures 1A, B**), supporting the use of zebrafish for functional analysis. We therefore established a zebrafish *vars2* knockdown model utilizing a *vars2* -targeted morpholino (MO). RT-qPCR confirmed that *vars2* expression was reduced by approximately 85% in *vars2* morphants (**Supplementary Figure 1C**).

Morphological assessment revealed concurrent developmental defects in skeletal muscle and spinal motor neurons following *vars2* depletion. Polarized light microscopy showed that while the standard control MO (STD MO) group displayed regularly arranged muscle fibers with distinct somite borders, whereas *vars2* morphants exhibited disorganized muscle fibers, a reduced somite count, and indistinct segment boundaries (**Figure 2A**). Analysis in *Tg(mylpfa:RFP)* transgenic zebrafish showed that the regular alignment and continuous fluorescent signals of fast muscle fibers observed in the STD MO group were disrupted in *vars2* morphants, which displayed reduced fluorescence intensity and disorganized muscle fibers (**Figure 2B**). In parallel, spinal motor neuron development was also impaired. Whole-mount *in situ* hybridization for isl1 showed well-formed spinal motor neurons with identifiable ganglia in the STD MO group at 72 hours post fertilization (hpf), whereas *vars2* morphants presented with disorganized motor neuron arrangements and fewer distinct ganglia (**Figure 2C**). *Tg(mnx1:EGFP)* transgenic larvae showed that the regular motor neuron branching present in the STD MO group was replaced by disorganized branching patterns and restricted axonal extension in the morphants (**Figure 2D**).

**Figure 2:**
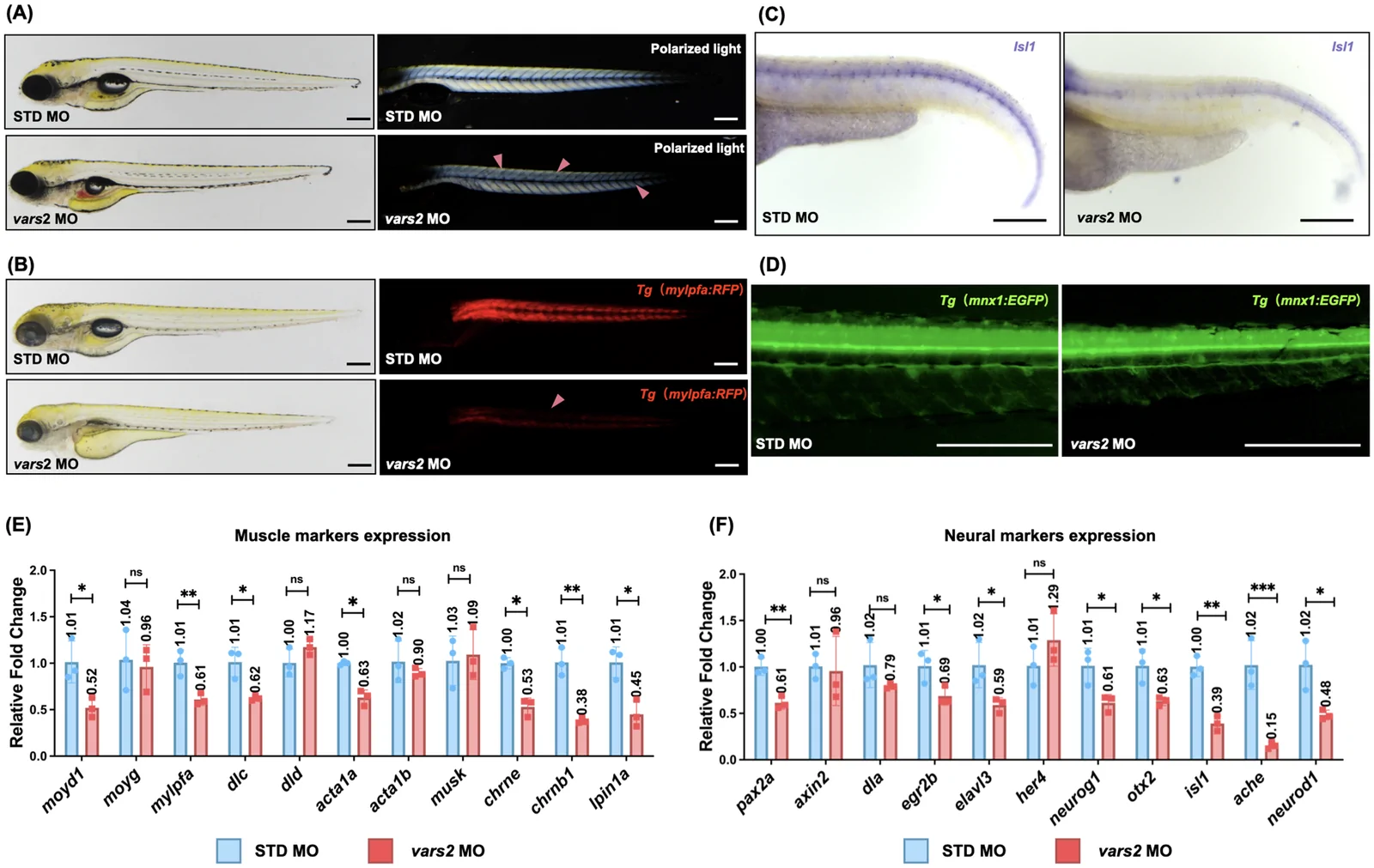
*vars2* depletion impairs skeletal muscle development and spinal motor neuron formation in zebrafish. **(A)** Bright-field and polarized light images of zebrafish larvae in the STD MO and *vars2* MO groups. **(B)** Fluorescence images of fast muscle fibers in *Tg(mylpfa:RFP)* larvae. **(C)** Whole-mount *in situ* hybridization assay for *isl1*. **(D)** Fluorescence images of spinal motor neurons in *Tg(mnx1:EGFP)* larvae. **(E)** RT-qPCR analysis of myogenic and neuromuscular junction-related genes. **(F)** RT-qPCR analysis of neural and neurogenesis-related genes. Scale bar, 250 μm. For RT-qPCR analysis, n = 3 biological replicates, with 20 embryos per biological replicate. Bar plots show individual biological replicates. *P < 0.05; **P < 0.01; ***P < 0.001; ns, not significant.

RT-qPCR analysis further showed broad downregulation of myogenic markers (*myod1*, *myog*, and *acta1a*) and NMJ-related genes (*musk*, *chrne*, and *chrnb1*) in *vars2* morphants (**Figure 2E**). Concurrently, essential neural and neurogenesis markers, including *pax2a*, *isl1*, *ache*, *neurog1*, and *neurod1*, were also significantly downregulated in the *vars2* morphants (**Figure 2F**). Together, these findings indicate that *vars2* is required for normal skeletal muscle and spinal motor neuron development in zebrafish, suggesting that its loss may impair neuromuscular function.

### 2.3 *vars2* deficiency induces an SNMG-like motor phenotype in zebrafish

Given the neuromuscular defects observed in *vars2* morphants, we next examined whether *vars2* deficiency also impaired motor function. At 5 day post fertilization (dpf), *vars2* morphants exhibited locomotor deficits, characterized by shortened swimming trajectories under basal conditions compared with the STD MO group (**Figures 3A–C**). Quantitative analysis showed that cumulative active movement time was reduced to 36% of control levels, whereas average swimming velocity decreased to 17% of control values (approximately 0.1 mm/s). In contrast, resting time was increased nearly four-fold in *vars2* morphants (**Figures 3D–F**).

**Figure 3:**
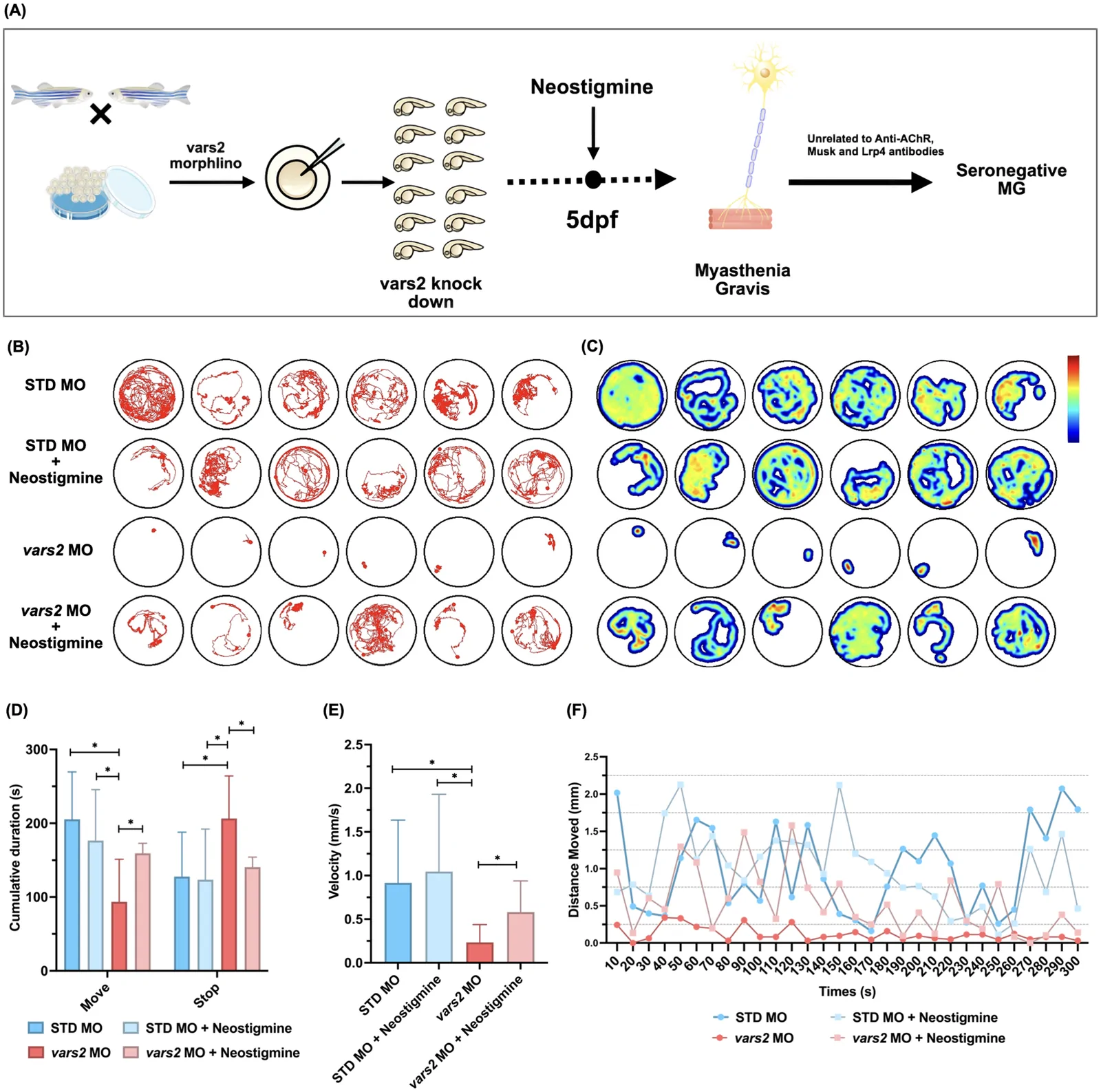
*vars2* deficiency induces a myasthenia-like motor phenotype in zebrafish that is responsive to neostigmine treatment. **(A)** Schematic diagram of the experimental design for behavioral analysis and neostigmine treatment in zebrafish larvae. **(B)** Representative swimming trajectories of larvae in the STD MO, vars2 MO, STD MO + neostigmine, and vars2 MO + neostigmine groups. **(C)** Representative movement heatmaps of larvae in the STD MO, vars2 MO, STD MO + neostigmine, and vars2 MO + neostigmine groups. **(D)** Quantification of cumulative active/stop movement time in each group. **(E)** Quantification of average swimming velocity in each group. **(F)** Quantification of total swimming distance in each group. For behavioral analysis, n = 6 biological replicates per group, with each individual zebrafish larvae was considered one biological replicate. Bar plots show individual biological replicates. *P < 0.05; **P < 0.01; ***P < 0.001; ns, not significant.

To determine whether this motor impairment was related to myasthenic dysfunction, we treated larvae with neostigmine, a canonical acetylcholinesterase inhibitor used in clinical MG management. Neostigmine significantly improved the locomotor abnormalities of *vars2* morphants, increasing cumulative movement time, swimming speed, and total travel distance, while reducing resting time (**Figures 3B−F**). These results indicate that *vars2* depletion induces an MG-like motor phenotype in zebrafish that is responsive to acetylcholinesterase inhibition.

### 2.4 Loss of *vars2* induces mitochondrial structural damage and oxidative stress in zebrafish embryos

To elucidate the cellular mechanisms underlying the observed motor deficits, and given that *VARS2* encodes a mitochondrial valyl-tRNA synthetase, we next examined whether the motor abnormalities in *vars2* morphants were accompanied by mitochondrial defects. MitoTracker staining showed an approximately 27% reduction in mitochondrial abundance in *vars2* morphants compared with the STD MO group (**Figures 4A, B**). Consistent with this, genes involved in mitochondrial fusion/fission dynamics (*mfn2*, *fis1*, *opa1*) and membrane transport (*timm23a*, *tomm20*) were significantly downregulated (**Figure 4C**).

**Figure 4:**
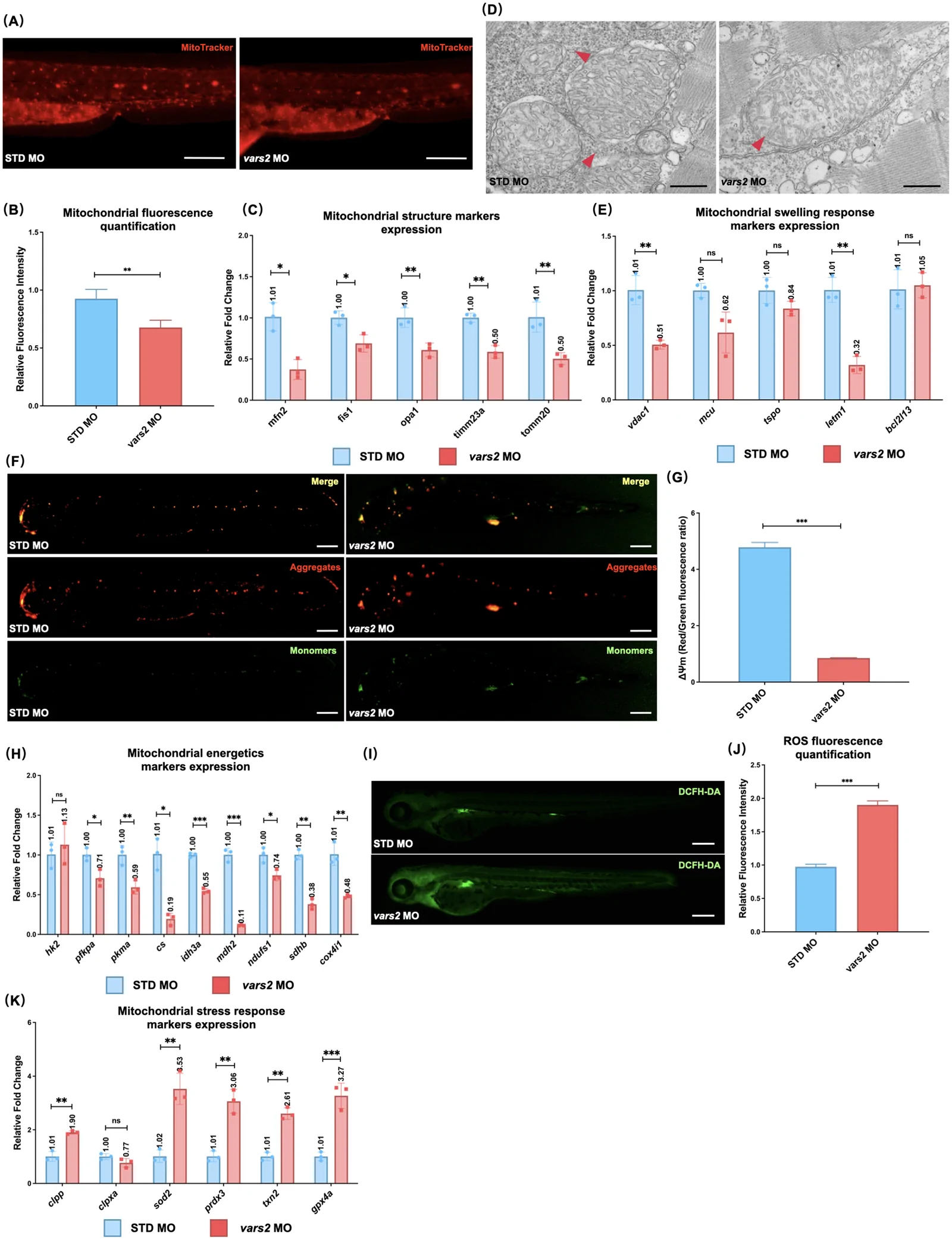
*vars2* deficiency disrupts mitochondrial structure and redox homeostasis in zebrafish embryos. **(A)** Representative MitoTracker staining images in zebrafish embryos from the STD MO and *vars2* MO groups. **(B)** Quantification of MitoTracker fluorescence intensity in the STD MO and *vars2* MO groups. **(C)** RT-qPCR analysis of genes related to mitochondrial fusion/fission dynamics and mitochondrial structure. **(D)** Representative transmission electron microscopy (TEM) images of skeletal muscle mitochondria in the STD MO and *vars2* MO groups. **(E)** RT-qPCR analysis of genes related to mitochondrial calcium handling and ion homeostasis. **(F)** Representative JC-1 staining images for mitochondrial membrane potential in the STD MO and *vars2* MO groups. **(G)** Quantification of the JC-1 red/green fluorescence ratio in the STD MO and vars2 MO groups. **(H)** RT-qPCR analysis of genes involved in glycolysis, the tricarboxylic acid (TCA) cycle, and oxidative phosphorylation (OXPHOS). **(I)** Representative DCFH-DA staining images for reactive oxygen species (ROS) in the STD MO and *vars2* MO groups. **(J)** Quantification of ROS fluorescence intensity in the STD MO and *vars2* MO groups. **(K)** RT-qPCR analysis of genes related to mitochondrial proteostasis and oxidative stress responses. Scale bars: 250 μm in **(A, F, I)**; 500 nm in **(D)** For RT-qPCR analysis, n = 3 biological replicates, with 20 embryos per biological replicate. Bar plots show individual biological replicates. *P < 0.05; **P < 0.01; ***P < 0.001; ns, not significant.

To resolve these defects at the subcellular level, we performed transmission electron microscopy (TEM) on skeletal muscle at 5 dpf. While the STD MO group muscle exhibited intact mitochondria with well-organized cristae, *vars2* morphants displayed pronounced ultrastructural abnormalities, including cristae disruption, matrix swelling, and irregular organelle morphology (**Figure 4D**). In parallel, genes involved in mitochondrial calcium handling (*vdac1*, *mcu*, *tspo*) and ion homeostasis (*letm1*, *bcl2l13*) were markedly dysregulated in *vars2* morphants (**Figure 4E**), further supporting impaired mitochondrial integrity.

Beyond structural abnormalities, *vars2* deficiency impaired mitochondrial functional and metabolic capacity. JC-1 staining showed a loss of mitochondrial membrane potential (Δψm) in *vars2* morphants (**Figure 4F**), and this depolarization was confirmed by the quantification of the red/green fluorescence ratio (**Figure 4G**). *vars2* depletion suppressed energy-generating pathways, indicated by the downregulation of genes involved in glycolysis (*hk2*, *pfkpa*, *pkma*), the tricarboxylic acid (TCA) cycle (*cs*, *idh3a*, *mdh2*) and OXPHOS (*ndufs1*, *sdhb*, *cox4i1*) (**Figure 4H**). Consistent with mitochondrial dysfunction, reactive oxygen species (ROS) accumulation was significantly increased in *vars2* morphants (**Figure 4I**), with quantification confirming this elevation (**Figure 4J**). Moreover, mitochondrial proteostasis genes (*clpp*, *clpxa*) were downregulated, whereas ROS-response genes (*sod2*, *prdx3*, *txn2*, *gpx4a*) were upregulated (**Figure 4K**). Together, these findings indicate that *vars2* is required to maintain mitochondrial structural and metabolic homeostasis in zebrafish.

### 2.5 *vars2* depletion induces global transcriptomic remodeling and immune dysregulation

To delineate the systemic molecular alterations driven by *vars2* deficiency, we performed RNA-seq on zebrafish larvae. GO and Reactome pathway analyses of the upregulated genes revealed significant enrichment in immune- and stress-related processes, including inflammatory activation, extracellular matrix remodeling, neutrophil degranulation, antigen presentation, and JAK-STAT signaling (**Figures 5A, A’**; **Supplementary Tables 11**, **12**). In contrast, downregulated transcripts were mainly enriched in pathways associated with synaptic and metabolic functions. Functional annotation of these repressed genes highlighted chemical synaptic transmission, synapse organization, monoatomic ion transport and channel activity, neuromuscular junction- and postsynaptic membrane-related components, as well as fatty acid metabolic and peroxisomal processes (**Figures 5B, B’**; **Supplementary Tables 11**, **12**). Together, these transcriptomic changes indicate broad disruption of neuro-immune and metabolic homeostasis in *vars2* morphants.

**Figure 5:**
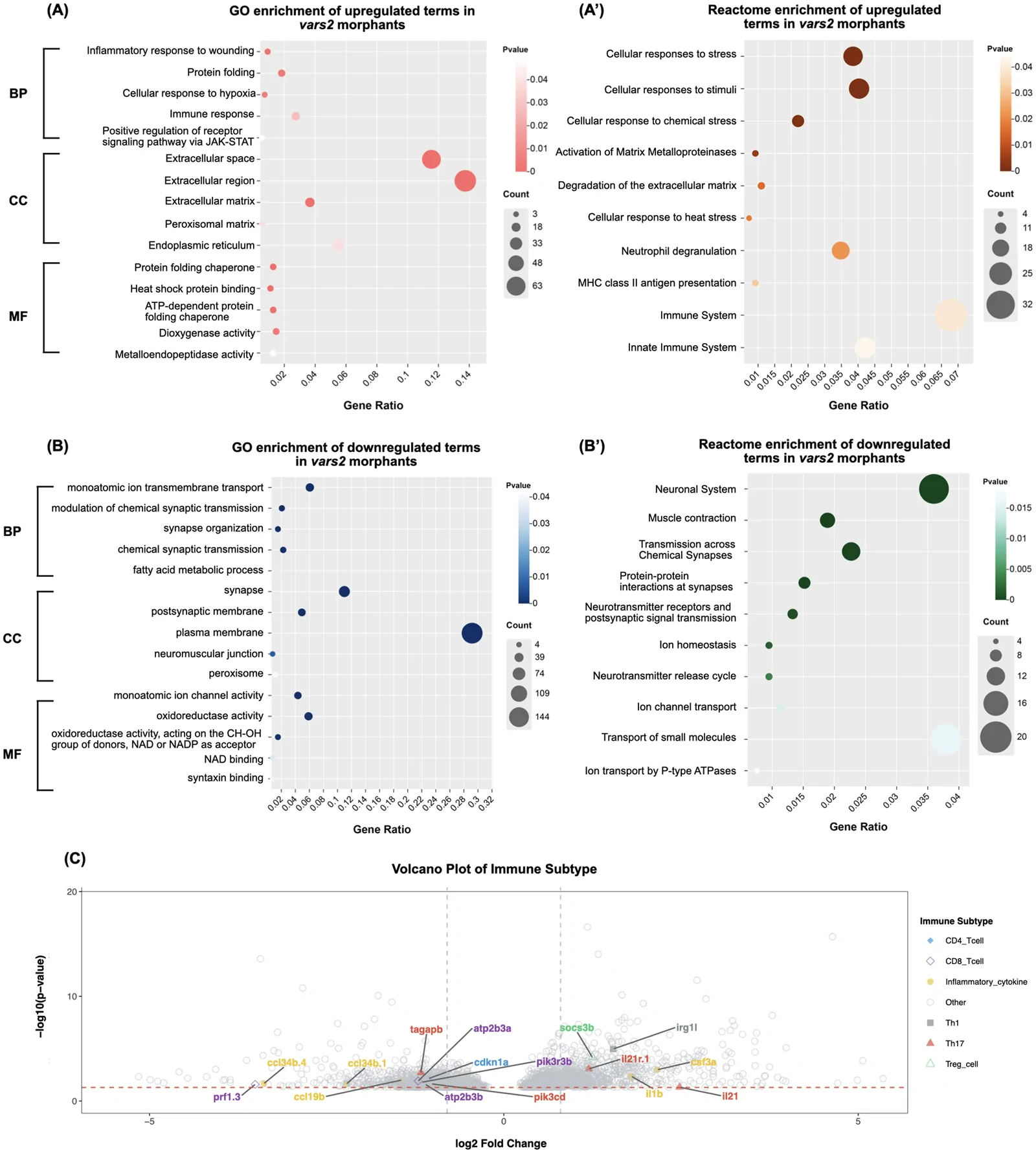
*vars2* depletion induces global transcriptomic remodeling and immune-related gene expression changes in zebrafish. **(A)** GO enrichment analysis of upregulated genes in the STD MO and vars2 MO groups. **(A′)** Reactome enrichment analysis of upregulated genes in the STD MO and *vars2* MO groups. **(B)** GO enrichment analysis of downregulated genes in the STD MO and *vars2* MO groups. **(B′)** Reactome enrichment analysis of downregulated genes in the STD MO and *vars2* MO groups. **(C)** Volcano plot showing differential expression of immune subtype-related genes in the STD MO and *vars2* MO groups.

Targeted analysis of immune-related differentially expressed genes further refined the nature of this inflammatory shift. Immune-associated transcripts displayed subtype-related alterations, with Th17-associated genes including *il1b*, *il21*, and *il17f* markedly upregulated in *vars2* morphants, whereas several transcripts related to CD8^+^ T cell function were downregulated (**Figure 5C**). This immune expression pattern supports a *vars2* deficiency-induced immunometabolic shift characterized by enhanced pro-inflammatory signaling and Th17-skewed immune activation, providing a molecular basis for the immune abnormalities observed in the SNMG-like model.

### 2.6 *vars2* deficiency drives immune imbalance characterized by T cell subset dysregulation and myeloid cell recruitment in zebrafish

To investigate the transcriptomic shift towards immune activation *in vivo*, we evaluated the immunological state following *vars2* depletion. Analysis of *Tg(rag2:mCherry)* larvae revealed thymic expansion and redistribution of *rag2*-positive T cell precursors in *vars2* morphants compared with the STD MO group (**Figure 6A**), suggesting altered T cell homeostasis. RT-qPCR analysis showed that CD4^+^ T cell-related genes (*cd4-1*, *lck*, *trac*) were significantly upregulated in *vars2* morphants (**Figure 6B**), whereas CD8^+^ T cell-related markers (*cd8a*, *ifng1*, *gzm3*) remained unaltered between groups (**Figure 6C**).

**Figure 6:**
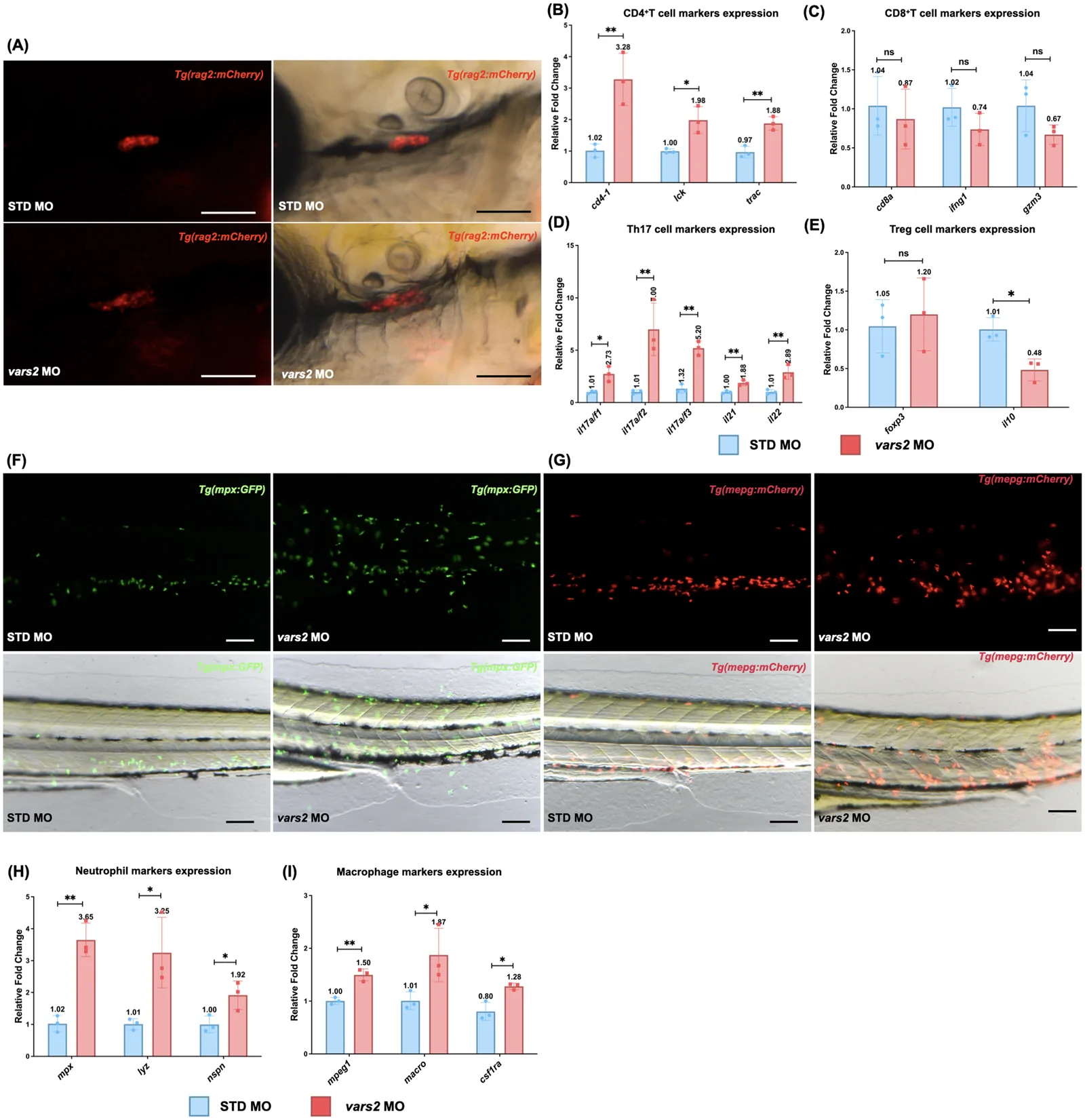
*vars2* deficiency induces immune imbalance characterized by T cell dysregulation and myeloid cell recruitment in zebrafish. **(A)** Representative fluorescence and merged bright-field images of *Tg(rag2:mCherry)* larvae in the STD MO and vars2 MO groups. **(B)** RT-qPCR analysis of CD4^+^ T cell marker genes in the STD MO and vars2 MO groups. **(C)** RT-qPCR analysis of CD8^+^ T cell-related genes in the STD MO and *vars2* MO groups. **(D)** RT-qPCR analysis of Th17-related genes in the STD MO and *vars2* MO groups. **(E)** RT-qPCR analysis of Treg-related genes in the STD MO and *vars2* MO groups. **(F)** Representative fluorescence images (upper panels) and corresponding merged bright-field images (lower panels) of neutrophils in *Tg(mpx1:GFP)* larvae from the STD MO and *vars2* MO groups. **(G)** Representative fluorescence images (upper panels) and corresponding merged bright-field images (lower panels) of macrophages in *Tg(mpeg:mCherry)* larvae from the STD MO and *vars2* MO groups. **(H)** RT-qPCR analysis of neutrophil marker genes in the STD MO and *vars2* MO groups. **(I)** RT-qPCR analysis of macrophage marker genes in the STD MO and *vars2* MO groups. Scale bars in **(A)**, **(F)**, and **(G)**, 100 μm. For RT-qPCR analysis, n = 3 biological replicates, with 20 embryos per biological replicate. Bar plots show individual biological replicates. *P < 0.05; **P < 0.01; ***P < 0.001; ns, not significant.

Subsequent evaluation of CD4^+^ T cell subset polarization revealed that the Th17-associated cytokines (*il17a/f1*, *il17a/f2*, *il17a/f3*, *il21*, and *il22*) were comprehensively upregulated (**Figure 6D**), whereas the Treg marker *foxp3* exhibited no significant change, and the immunosuppressive cytokine *il10* was significantly downregulated (**Figure 6E**).

We further examined the dynamic changes of neutrophils and macrophages, given that myeloid immune cells are important effector cells in local inflammatory responses and tissue damage. *In vivo* tracking utilizing *Tg(mpx1:GFP)* transgenic larvae demonstrated enhanced recruitment and the spatial distribution of neutrophils in *vars2* morphants relative to the STD MO group (**Figure 6F**). Similarly, *Tg(mpeg:mCherry)* transgenic larvae analysis showed macrophages evenly distributed in the STD MO group skeletal muscle, but markedly accumulated in structurally disorganized tissue regions in *vars2* morphants (**Figure 6G**). These morphological observations were further supported by RT-qPCR analysis showing significant upregulation of neutrophil markers (*mpx*, *lyz*, and *nspn*) (**Figure 6H**) and macrophage markers (*mpeg1*, *macro*, and *csf1ra*) (**Figure 6I**) in the *vars2* morphants. Together, these results indicate that *vars2* deficiency establishes a highly inflammatory *in vivo* microenvironment, linking T cell imbalance and local accumulation of activated myeloid cells.

### 2.7 Pharmacological interventions targeting mitochondrial, neuromuscular, and immune axes ameliorate *vars2* depletion-induced deficits

To further investigate the functional relationships among mitochondrial dysfunction, immune dysregulation, and neuromuscular impairment, we evaluated mechanism-specific pharmacological interventions in *vars2* morphants. For upstream targets, we administered CoQ10 (to restore mitochondrial function) and neostigmine (to enhance neuromuscular transmission). Locomotor trajectory analysis and kinematic heatmaps showed that the severely reduced swimming activity in *vars2* morphants was partially restored by individual treatment with either CoQ10 or neostigmine (**Figures 7A, B**). Notably, co-administration of both agents yielded an enhanced therapeutic efficacy. RT-qPCR analysis showed that the elevated expression of CD4^+^/Th17-associated transcripts (*cd4-1*, *lck*, *trac*, *il17a/f1*, *il17a/f2*, *il17a/f3*, *il21*, and *il22*) and myeloid-associated markers (*mpx*, *lyz*, *nspn*, *mpeg1*, *macro*, and *csf1ra*), alongside the suppressed Treg marker *il10*, were partially reversed by treatment with CoQ10 or neostigmine alone, and the recovery was more marked under the combined treatment, while CD8^+^ T cell markers (*cd8a*, *ifng1*, and *gzm3*) remained relatively stable across conditions (**Figures 7C−H**).

**Figure 7:**
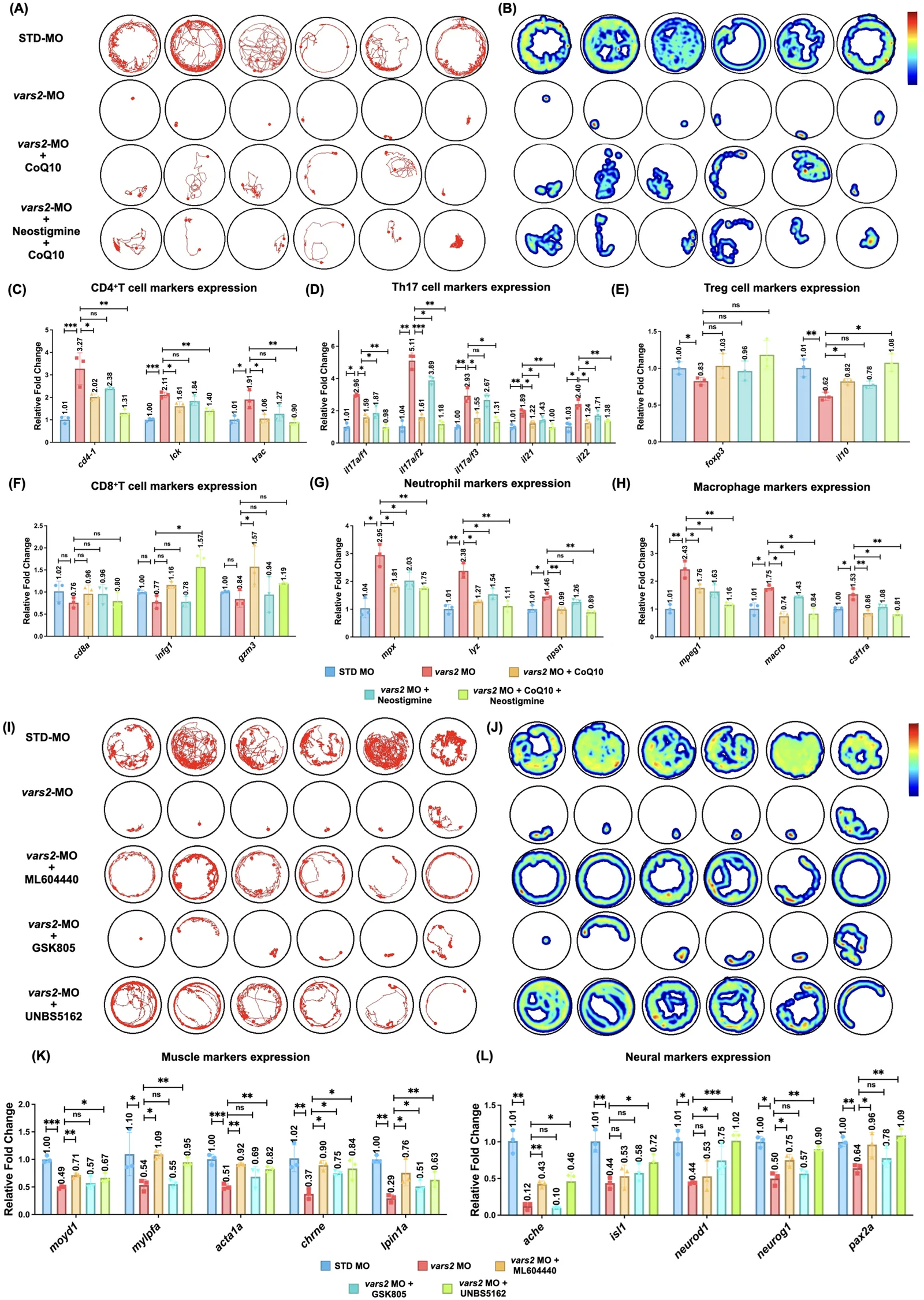
Pharmacological interventions targeting mitochondrial, neuromuscular, and immune axes ameliorate vars2 depletion-induced deficits. **(A–H)** larvae were assigned to the STD MO, *vars2* MO, *vars2* MO + CoQ10, *vars2* MO + neostigmine, and *vars2* MO + CoQ10 + neostigmine groups. **(A)** Representative swimming trajectories. **(B)** Representative movement heatmaps. **(C)** RT-qPCR analysis of CD4^+^ T cell-related genes. **(D)** RT-qPCR analysis of CD8^+^ T cell-related genes. **(E)** RT-qPCR analysis of Th17-related genes. **(F)** RT-qPCR analysis of Treg-related genes. **(G)** RT-qPCR analysis of neutrophil-related genes. **(H)** RT-qPCR analysis of macrophage-related genes. **(I–L)** larvae were assigned to the STD MO, *vars2* MO, *vars2* MO + ML604440, *vars2* MO + GSK805, and *vars2* MO + UNBS5162 groups. **(I)** Representative swimming trajectories. **(J)** Representative movement heatmaps. **(K)** RT-qPCR analysis of myogenic genes. **(L)** RT-qPCR analysis of neurogenic genes. For RT-qPCR analysis, n = 3 biological replicates, with 20 embryos per biological replicate. For behavioral analysis, n = 6 biological replicates per group, with each individual zebrafish larvae was considered one biological replicate. Bar plots show individual biological replicates. *P < 0.05; **P < 0.01; ***P < 0.001; ns, not significant.

These experiments were designed to assess whether interventions targeting upstream mitochondrial or neuromuscular abnormalities could also alleviate the associated immune dysregulation. Conversely, we next asked whether directly suppressing the downstream immune abnormalities could improve the neuromuscular phenotype. We subsequently investigated whether directly targeting the downstream immune dysregulation could mitigate the disease phenotype utilizing specific immunomodulatory agents ML604440 (proteasome β1i/LMP2 inhibitor, blocking naïve T cell differentiation into Th17 cells), GSK805 (RORγt inhibitor, suppressing Th17 cell function), and UNBS5162 (broad-spectrum CXCL antagonist, inhibiting excessive myeloid cell attack). All three immune-targeted treatments effectively ameliorated the locomotor deficits in *vars2* morphants (**Figures 7I, J**). Consistent with these behavioral improvements, the abnormal expression of critical myogenic (*myod1*, *mylpfa*, *acta1a*, *chrne*, and *lpin1*) and neurogenic (*ache*, *isl1*, *neurod1*, *neurog1*, and *pax2a*) markers was substantially restored across the treatment groups (**Figures 7K, L**). Collectively, these pharmacological rescue experiments demonstrate that intervening at the level of mitochondrial function, neuromuscular transmission, or downstream immune signaling can effectively alleviate the *vars2* deficiency-induced myasthenic pathology, thereby functionally validating the integrated neuro-immune-metabolic mechanism underlying SNMG.

## 3 Discussion

By integrating genetic association analysis, single-cell transcriptomics, and *in vivo* functional evaluation, this study identifies VARS2 as a candidate contributor to SNMG and suggests that its pathogenic relevance lies in linking mitochondrial dysfunction to immune and neuromuscular abnormalities. Beyond its established role in mitochondrial translation, VARS2 may participate in an SNMG-related immunometabolic axis centered on CD4+ T cell dysregulation. In zebrafish, vars2 deficiency caused coordinated defects in mitochondrial integrity, immune homeostasis, and neuromuscular development, resulting in a myasthenia-like motor phenotype that was partially rescued by mitochondrial support, acetylcholinesterase inhibition, and immune modulation. Across the RT-qPCR datasets, 2−ΔCt-based analyses further contextualized the observed fold changes by revealing substantial differences in basal expression abundance among the examined genes (**Supplementary Figures 1**–**S5**). The comparable β-actin Ct values between STD MO and vars2 MO groups across experiments further supported its stability as the reference gene (**Supplementary Figure 6**). Based on these findings, we present a mechanistic framework in which VARS2 insufficiency promotes mitochondrial dysfunction, CD4+ T cell-associated immune imbalance, and downstream neuromuscular injury, thereby contributing to SNMG pathogenesis (**Figure 8**).

**Figure 8:**
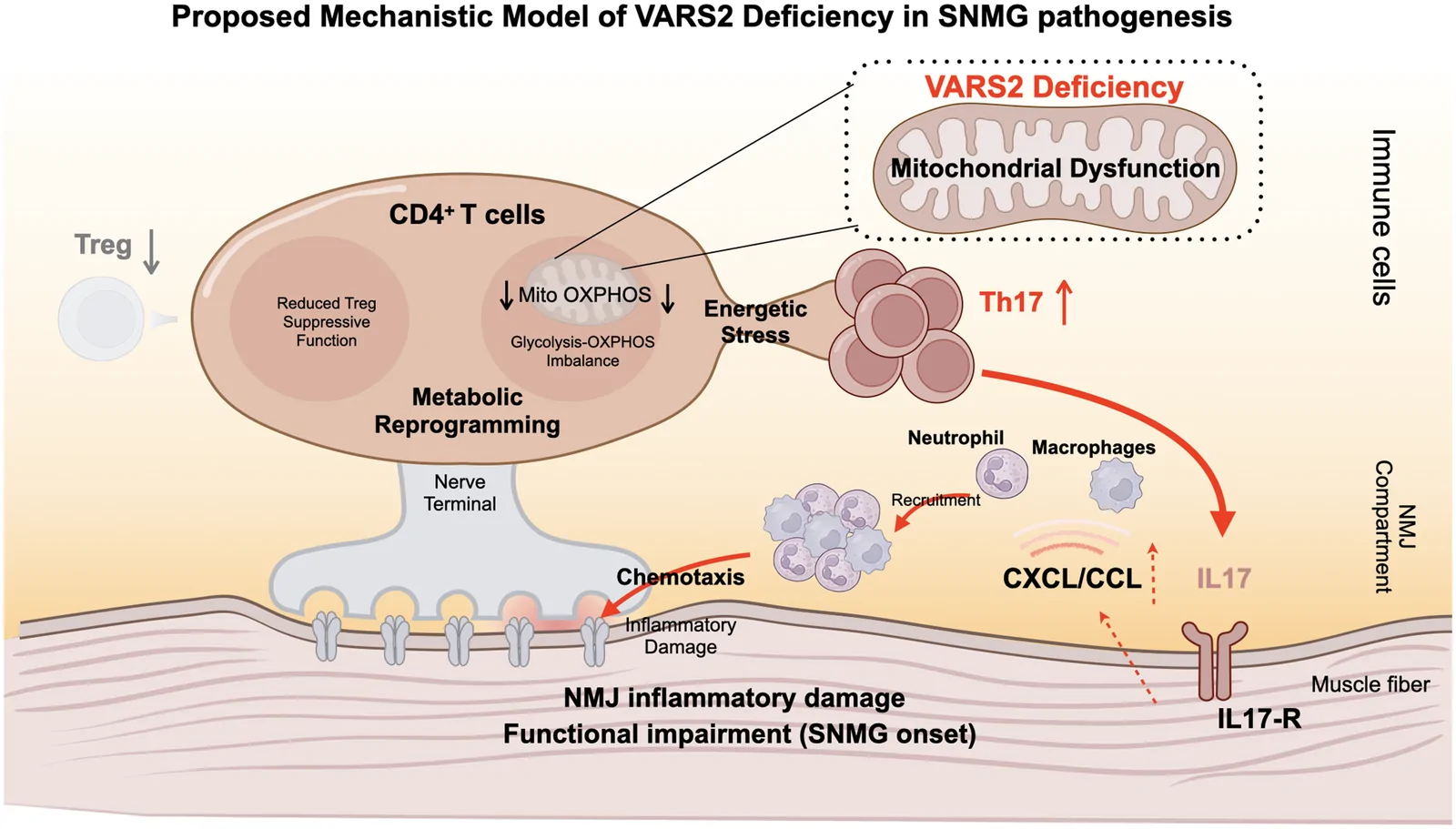
Mechanistic framework linking VARS2 deficiency to mitochondrial, immune, and neuromuscular dysregulation in SNMG.

One important issue raised by our data is the apparent discrepancy between the genetic association analysis and the single-cell expression results. The SMR analysis captures the relationship between genetically regulated *VARS2* expression and overall MG susceptibility, whereas the single-cell data reflect disease-state transcriptional changes within specific immune-cell subsets. These readouts are therefore not directly equivalent. Moreover, the single-cell findings should be interpreted in the context of the source cohort: the SNMG patients had refractory early-onset generalized MG, received long-term immunosuppressive treatment, and were sampled before IVIg rather than before any immunotherapy (18). Seronegative status was also defined without live cell-based assays, so low-titer or low-affinity autoantibodies cannot be excluded (19). These factors limit subtype-specific and causal interpretation of the transcriptomic differences. Nevertheless, the increased *VARS2* expression in naïve CD4+ T cells may reflect disease-state or compensatory transcriptional remodeling rather than the primary direction of pathogenic effect (20, 21). Consistent with this interpretation, *in vivo* vars2 knockdown induced locomotor and neuromuscular abnormalities that were partially rescued by neostigmine. Together with the genetic association results, these functional data support a protective role for VARS2 in maintaining neuromuscular integrity, while its specific relevance to rigorously defined SNMG warrants further validation in independent, treatment-matched, and appropriately serotyped cohorts.

Our findings also extend current views of MG immunopathology. Previous studies have identified broad immune dysregulation in MG beyond autoantibody production, including altered CD4^+^ T cell activation, Th17/Treg imbalance, inflammatory signaling abnormalities, and disrupted Tfh/Tfr-associated immune organization (22–27). However, these alterations have mainly been described at the level of immune phenotypes and cytokine imbalance, with limited evidence linking them to defined mitochondrial mechanisms. Our data suggest that, in SNMG, immune dysregulation may be mechanistically coupled to mitochondrial dysfunction. *VARS2* dysregulation was most evident in CD4^+^ T cells from SNMG patients, and *vars2* deficiency *in vivo* was associated with mitochondrial dysfunction, ROS accumulation, metabolic suppression, and a Th17-dominant inflammatory profile with reduced *il10* expression. Together, these findings support mitochondrial dysfunction as a potential upstream driver of immune imbalance in SNMG.

This interpretation is biologically plausible in light of the known role of *VARS2* and the metabolic sensitivity of T cell fate decisions. *VARS2* encodes the mitochondrial valyl-tRNA synthetase and is required for mitochondrial translation, respiratory-chain assembly, and OXPHOS homeostasis (12, 13). Previous studies of *VARS2* have mainly focused on primary mitochondrial disorders and multisystem energetic failure (14, 15, 28), whereas its role in immune regulation has remained largely unexplored. In parallel, CD4^+^ T cell activation and differentiation are highly dependent on mitochondrial bioenergetics, redox control, and metabolic reprogramming, with the Th17/Treg axis being especially sensitive to such perturbations (11, 28–30). Our data connect these previously separate lines of evidence by suggesting that *VARS2* dysfunction may promote SNMG-related immune remodeling through disruption of mitochondrial homeostasis.

Beyond adaptive immunity, our results also implicate innate immune recruitment in the pathological consequences of *vars2* deficiency. The increase in neutrophils and macrophages in the zebrafish model is consistent with growing evidence that innate immune pathways contribute to chronic inflammatory remodeling, thymic microenvironmental alteration, and tissue injury in MG (29, 31, 32). These findings indicate that *VARS2* dysfunction not only perturbs adaptive immune regulation but also promotes innate immune recruitment, thereby reinforcing local inflammatory injury at the tissue level. Our data therefore support an integrated pathogenic framework in which mitochondrial stress, adaptive immune deviation, and innate immune activation are mechanistically linked rather than operating as isolated processes.

The intervention experiments further corroborate this framework. *In vivo* modulation of mitochondrial dysfunction, neuromuscular transmission, and downstream inflammatory pathways each partially alleviated the *vars2*-deficient phenotype. In particular, immune-targeted interventions directed at naïve T cell differentiation into Th17 cells, Th17-associated effector responses, and chemokine-related signaling improved locomotor phenotypes and corrected neural and muscular molecular abnormalities, consistent with prior evidence implicating inflammatory and CXCL-associated pathways in MG immune remodeling (22–24, 33, 34). In parallel, upstream intervention with CoQ10 and neostigmine also ameliorated the defects induced by *vars2* deficiency, suggesting that mitochondrial support and restoration of cholinergic neuromuscular transmission can independently modify the disease phenotype. However, because the pharmacological experiments were designed to interrogate different levels of the proposed pathogenic pathway, reciprocal assessment of both immune and neuromuscular markers was not performed for every intervention. Future studies incorporating such reciprocal analyses will be important to more comprehensively define the relationships among these pathological processes. Taken together, these findings support a multilayer pathogenic model in which mitochondrial impairment, immune dysregulation, and neuromuscular dysfunction act in concert to drive an SNMG-relevant myasthenic phenotype.

In summary, our study identifies a potential link between *VARS2* deficiency, mitochondrial dysfunction, and CD4^+^ T cell-related immune disturbance in SNMG, offering a new perspective on the interplay between immune imbalance and neuromuscular injury. Although the present findings support a role for *VARS2* in SNMG, several important questions remain to be resolved. Further studies in independent SNMG cohorts and complementary experimental models will be needed to validate the disease relevance of VARS2 and to define its role in long-term pathological progression. In particular, it remains unclear whether VARS2 directly regulates metabolic programming and lineage differentiation within CD4^+^ T cells, or whether its effects occur indirectly through broader mitochondrial stress and changes in the immune microenvironment. Future studies combining purified immune cell subsets, cell type-specific models, and patient-derived samples will be needed to address this question. Such work may clarify how mitochondrial dysfunction contributes to immune imbalance and neuromuscular injury in SNMG, and may also inform the development of mechanism-based therapies.

## 4 Materials and methods

### 4.1 Data source

Publicly available datasets were used in this study. Whole-blood *cis*-eQTL data were obtained from the eQTLGen Consortium, which included 31,684 blood samples (35). GTEx v8 data were used as an additional eQTL resource where needed (36). GWAS summary statistics for MG were obtained from two public datasets: the study by Chia R et al. (1,873 cases and 36,370 controls) and the FinnGen R10 release (461 cases and 408,430 controls) (37, 38).

Single-cell RNA-sequencing (scRNA-seq) data were obtained from the public dataset GSE227835, generated on the DNBSEQ-G400 platform (GPL28038) (18). This dataset included healthy controls, AChR-positive MG patients, and SNMG patients. In the present study, only healthy controls, AChR-positive MG patients, and SNMG patients were included. Detailed information on the included studies and consortia is provided in **Supplementary Table 1**.

### 4.2 Genetic and single-cell analyses

MG GWAS summary statistics were quality controlled by removing variants with missing values, invalid statistics, duplicated SNP identifiers, or minor allele frequency < 0.5%. Meta-analysis was performed using METAL based on the standard error scheme (39). SMR analysis was conducted using SMR software (version 1.3.1) with a *cis*-window of 1 Mb. Genome-wide significant *cis*-eQTLs (*P* < 5 × 10^-8^) were retained as instrumental variants, and linkage disequilibrium clumping was performed using the European reference panel from the 1000 Genomes Project (40). Multiple testing correction was carried out using the p.adjust function in R, and genes with FDR-adjusted *P* < 0.05 were considered significant (40). The full SMR results are provided in **Supplementary Table 2**. HEIDI tests were used to assess heterogeneity, and colocalization analysis was performed using the coloc R package (version 5.2.3), with PPH_4_ ≥ 0.8 considered strong evidence of colocalization (41, 42). Candidate genes shared by genetic and single-cell transcriptomic analyses are listed in **Supplementary Table 8**.

For single-cell analysis, genes detected in fewer than three cells were excluded. Cells with fewer than 200 or more than 4,000 detected genes, or with mitochondrial gene content >20%, were excluded from downstream analysis. After filtering, 94,920 cells and 73,917 genes were retained (43). Data were analyzed using Seurat (44). Expression data were normalized and scaled, followed by principal component analysis and Harmony-based batch correction. Cell clustering and visualization were performed using standard Seurat workflows (44), and cell types were annotated using the ScType database together with canonical immune markers (45). Marker genes distinguishing naïve CD4^+^ T cells from other immune cell populations are provided in **Supplementary Table 3.** Expression of target genes was compared among healthy controls, AChR-positive MG, and SNMG samples. Differential expression results for immune cells and naïve CD4+ T cells in the pairwise comparisons of SNMG versus healthy controls and SNMG versus AChR-positive MG are provided in **Supplementary Tables 4**–**7**.

### 4.3 Cross-species conservation analysis of VARS2

The full-length amino acid sequences of the VARS2 protein from seven vertebrate species were retrieved from the NCBI GenBank database. Multiple sequence alignment was performed using MultAlin. Structural models of VARS2 in human, mouse, and zebrafish were generated using AlphaFold3 to assess sequence and structural conservation.

### 4.4 Zebrafish maintenance and *vars2* knockdown

Adult zebrafish (AB strain) and the transgenic lines *Tg(mylpfa:RFP)*, *Tg(mnx1:EGFP)*, *Tg(rag2:mCherry)*, *Tg(mpx1:GFP)*, and *Tg(mpeg:mCherry)* were maintained at 28.5 °C under a 14 h light/10 h dark cycle. Embryos were obtained by natural mating and staged according to hours post fertilization or days post fertilization (46). All animal procedures were approved by the Experimental Animal Management and Ethics Committee of Northwest University (NWU-AWC-20241107Z).

Antisense morpholinos targeting zebrafish *vars2* (splice-donor morpholino, *vars2* MO) and a standard control morpholino (STD MO) were purchased from Gene Tools, LLC (USA) (**Supplementary Table 9**). Morpholinos were injected at the 1-cell stage to generate transient vars2 knockdown embryos. Knockdown efficiency was assessed by RT-qPCR at 24 hpf.

### 4.5 Behavioral and neuromuscular analyses

Locomotor behavior was assessed using the ZebraMonitor 2.0 system and ZebraEthogram 2.0 software (Huaxin Microfish, China). Larvae were acclimated at 28.5 °C for 30 min before recording. Locomotor activity was monitored for 30 min with a sampling interval of 10 s. Parameters including movement trajectory, total movement duration, resting time, swimming speed, and total swimming distance were analyzed. Heatmaps were generated to visualize activity intensity (47).

For morphological evaluation of the neuromuscular system, embryos at 72 hpf were examined by birefringence using an SMZ25 stereomicroscope with a DS-Ri2 digital camera (Nikon, Japan) to assess muscle integrity, including somite number and muscle fiber organization. *Tg(mylpfa:RFP)* and *Tg(mnx1:EGFP)* larvae were imaged using the same system to evaluate fast muscle fibers and motor neuron morphology. Whole-mount *in situ* hybridization was performed using a digoxigenin-labeled *isl1* probe (Roche Diagnostics, USA) to detect spinal motor neurons, with image acquisition performed as described above. The expression of key myogenic and neurogenic marker genes was quantified via RT-qPCR.

### 4.6 Assessment of mitochondrial structure and function

For ultrastructural examination of mitochondria, embryos at 48 hpf were fixed overnight at 4 °C in 2.5% glutaraldehyde prepared in 0.1 M phosphate buffer (pH 7.4), followed by post-fixation in 1% osmium tetroxide. Samples were dehydrated, embedded in Epon 812 resin, sectioned at 70 nm, and examined by TEM (Hitachi, Tokyo, Japan). The integrity of mitochondrial cristae and structural morphology were evaluated in randomly selected microscopic fields.

Mitochondrial staining intensity, ROS, and mitochondrial membrane potential were assessed by fluorescent staining. Embryos were stained with 1 μM MitoTracker™ Red CMXRos (Invitrogen, USA) for mitochondrial staining intensity, 10 μM DCFH-DA (Beyotime, China) for ROS detection, and 5 μM JC-1 (Sigma-Aldrich, USA) for mitochondrial membrane potential analysis. Fluorescence images were acquired using an SMZ25 stereomicroscope with a DS-Ri2 digital camera (Nikon, Japan) and quantified with ImageJ (version 1.8.0) (48). The transcript levels of genes regulating mitochondrial dynamics and oxidative stress responses were also measured.

### 4.7 RT-qPCR analysis

Total RNA was isolated from zebrafish embryos in each group using TRIzol reagent (Ambion, USA). cDNA was synthesized using SuperScript III (Invitrogen, USA) according to the manufacturer’s instructions. RT-qPCR was performed using the ChamQ Blue Universal SYBR RT-qPCR Master Mix (Vazyme, China) on a QuantGene 9600 Real-Time PCR System (Bioer Technology, China). Primer sequences are listed in **Supplementary Table 10**. *β-actin* was used as the internal reference, and relative expression levels were calculated using the 2^-ΔΔCt^ method (49).

### 4.8 RNA library preparation, sequencing and transcriptomic analysis in zebrafish

Total RNA was extracted from 5 dpf larvae in the STD MO and *vars2* MO groups using TRIzol reagent (Ambion, USA). RNA integrity was assessed using an Agilent 2100 Bioanalyzer, and samples with an RNA integrity number > 8.0 were used for library construction. Libraries were prepared using the TruSeq Stranded mRNA Kit (Illumina) and sequenced on the NovaSeq 6000 platform.

Raw reads were filtered using Trimmomatic (version 0.39) and aligned to the danRer11 reference genome using HISAT2 (version 2.2.1). Gene expression levels were quantified using featureCounts (version 2.0.1). Differentially expressed genes were identified using DESeq2 with |log2 fold change| > 1 and adjusted *P* < 0.05 as the cutoff values. Gene Ontology and Reactome enrichment analyses were performed using clusterProfiler (version 4.2.2).

### 4.9 Immune analyses in zebrafish

To assess immune alterations in *vars2* deficient zebrafish, *Tg(rag2:mCherry*), *Tg(mpx1:GFP)*, and *Tg(mpeg:mCherry)* larvae were imaged using an SMZ25 stereomicroscope with a DS-Ri2 digital camera (Nikon, Japan) to evaluate T cell precursors, neutrophils, and macrophages, respectively.

### 4.10 Pharmacological intervention experiments

To assess functional rescue, *vars2* MO larvae were treated with selected pharmacological agents targeting specific mechanistic axes. To evaluate mitochondrial and neuromuscular pathways, larvae were exposed to 50 μM coenzyme Q10 and 10 μM neostigmine, administered either individually or in combination. For immune intervention, larvae were treated with ML604440, GSK805, or UNBS5162 (TargetMol, China). Untreated STD MO larvae and vehicle-treated *vars2* MO larvae served as appropriate controls. Pharmacological exposure was initiated at 3 dpf, and samples were collected or analyzed at 5 dpf.

### 4.11 Statistical analysis

Sample sizes and biological replicates are specified in the corresponding figure legends. For RT-qPCR analysis, n = 3 biological replicates, with 20 embryos per replicate; for behavioral analysis, n = 6 larvae per group. Data are presented as the mean ± SD. For RT-qPCR data, a formal normality test was not performed because each group contained only three biological replicates, for which normality testing has very limited power. Statistical comparisons of RT-qPCR data were performed on ΔCt values using Welch’s two-tailed unpaired t-test, whereas relative expression was calculated using the 2^−ΔΔCt method for visualization. For other analyses with an adequate sample size, data distribution was assessed using the Shapiro–Wilk test. Multiple-group comparisons were performed using one-way ANOVA followed by Duncan’s multiple range test, or two-way ANOVA where appropriate. Results based on n = 3 biological replicates should be interpreted with appropriate caution. P < 0.05 was considered statistically significant.

## Data Availability

The raw sequence data reported in this paper have been deposited in the Genome Sequence Archive in National Genomics Data Center, China National Center for Bioinformation/Beijing Institute of Genomics, Chinese Academy of Sciences (GSA: CRA041728) that are publicly accessible at https://ngdc.cncb.ac.cn/gsa/browse/CRA041728.

## Supplementary material

Supplementary Figure 1 VARS2 is conserved across vertebrates and efficiently knocked down in zebrafish. **(A)** Multiple sequence alignment of VARS2 protein sequences from representative vertebrate species. **(B)** Predicted protein structures of VARS2 in human, mouse, and zebrafish. **(C)** RT-qPCR analysis of *vars2* expression in zebrafish embryos injected with STD MO or *vars2* MO. **(C′)** Relative quantification of *vars2* expression in the same samples, calculated as 2^−ΔCT and plotted on the indicated scale. For RT-qPCR analysis, n = 3 biological replicates, with 20 embryos per biological replicate. Bar plots show individual biological replicates. **P* < 0.05; ***P* < 0.01; ****P* < 0.001; ns, not significant.

Supplementary Figure 2 Relative quantification of muscle- and neural-related gene expression in vars2-depleted zebrafish. **(A)** Relative quantification (2^−ΔCt) of muscle- and neuromuscular junction-related genes in zebrafish embryos injected with STD MO or *vars2* MO. **(B)** Relative quantification (2^−ΔCt) of neural and neurogenesis-related genes in the STD MO and *vars2* MO groups. For RT-qPCR analysis, n = 3 biological replicates, with 20 embryos per biological replicate. Bar plots show individual biological replicates. **P* < 0.05; ***P* < 0.01; ****P* < 0.001; ns, not significant.

Supplementary Figure 3 Relative quantification of mitochondrial and metabolic gene expression in vars2-deficient zebrafish. **(A)** Relative quantification (2^−ΔCt) of genes related to mitochondrial structure in zebrafish embryos injected with STD MO or *vars2* MO. **(B)** Relative quantification (2^−ΔCt) of genes related to the mitochondrial swelling response in the STD MO and *vars2MO* groups. **(C)** Relative quantification (2^−ΔCt) of genes related to mitochondrial energetics in the STD MO and *vars2* MO groups. **(D)** Relative quantification (2^−ΔCt) of genes related to the mitochondrial stress response in the STD MO and *vars2* MO groups. For RT-qPCR analysis, n = 3 biological replicates, with 20 embryos per biological replicate. Bar plots show individual biological replicates. *P < 0.05; **P < 0.01; ***P < 0.001; ns, not significant.

Supplementary Figure 4 Relative quantification of immune-related gene expression in vars2-deficient zebrafish. **(A)** Relative quantification (2^−ΔCt) of CD4+ T cell marker genes in zebrafish embryos injected with STD MO or *vars2* MO. **(B)** Relative quantification (2^−ΔCt) of CD8+ T cell marker genes in the STD MO and *vars2* MO groups. **(C)** Relative quantification (2^−ΔCt) of Th17-related genes in the STD MO and *vars2* MO groups. **(D)** Relative quantification (2^−ΔCt) of Treg-related genes in the STD MO and *vars2* MO groups. **(E)** Relative quantification (2^−ΔCt) of neutrophil marker genes in the STD MO and *vars2* MO groups. **(F)** Relative quantification (2^−ΔCt) of macrophage marker genes in the STD MO and *vars2* MO groups. For RT-qPCR analysis, n = 3 biological replicates, with 20 embryos per biological replicate. *P < 0.05; **P < 0.01; ***P < 0.001; ns, not significant.

Supplementary Figure 5 Relative quantification of immune-, muscle-, and neural-related gene expression following pharmacological interventions in vars2-deficient zebrafish. **(A–F)** Relative quantification (2^−ΔCt) of (A) CD4^+^ T cell, **(B)** CD8^+^ T cell, **(C)** Th17, **(D)** Treg, **(E)** neutrophil, and **(F)** macrophage marker genes in the STD MO, *vars2* MO, *vars2* MO + CoQ10, *vars2* MO + neostigmine, and *vars2* MO + CoQ10 + neostigmine groups. **(G, H)** Relative quantification (2^−ΔCt) of **(G)** muscle-related and (H) neural-related genes in the STD MO, *vars2* MO, *vars2* MO + ML604440, *vars2* MO + GSK805, and *vars2* MO + UNBS5162 groups. For RT-qPCR analysis, n = 3 biological replicates, with 20 embryos per biological replicate. *P < 0.05; **P < 0.01; ***P < 0.001; ns, not significant.

Supplementary Figure 6 Stability of β-actin expression across RT-qPCR in STD MO and *vars2* MO groups. **(A)** Raw β-actin Ct values across all RT-qPCR experiments in the STD MO and *vars2* MO groups. Individual values and mean ± SD are shown (*P* = 0.803). **(B)** Paired comparison of mean β-actin Ct values (paired t-test, P = 0.681). ns, not significant.

## References

[1] Gilhus NE . Myasthenia gravis. N Engl J Med. (2016) 375:2570–81. doi: 10.1056/nejmra1602678 28029925

[2] Gilhus NE Verschuuren JJ . Myasthenia gravis: subgroup classification and therapeutic strategies. Lancet Neurol. (2015) 14:1023–36. doi: 10.1016/s1474-4422(15)00145-3 26376969

[3] Vinciguerra C Bevilacqua L Lupica A Ginanneschi F Piscosquito G Rini N . Diagnosis and management of seronegative myasthenia gravis: lights and shadows. Brain Sci. (2023) 13:1286. doi: 10.3390/brainsci13091286 37759888 PMC10526522

[4] Evoli A Palace J Spagni G Cheli M Ruiter A Verschuuren J . 275th ENMC international workshop: seronegative myasthenia gravis: an update paradigm for diagnosis and management, 9–11 February 2024, Hoofddorp, the Netherlands. Neuromuscul Disord. (2024) 44:104468. doi: 10.1016/j.nmd.2024.104468 39427494

[5] Uzawa A Kuwabara S Suzuki S Imai T Murai H Ozawa Y . Roles of cytokines and T cells in the pathogenesis of myasthenia gravis. Clin Exp Immunol. (2021) 203:366–74. doi: 10.1111/cei.13546 33184844 PMC7874834

[6] Masuda M Matsumoto M Tanaka S Nakajima K Yamada N Ido N . Clinical implication of peripheral CD4+CD25+ regulatory T cells and Th17 cells in myasthenia gravis patients. J Neuroimmunol. (2010) 225:123–31. doi: 10.1016/j.jneuroim.2010.03.016 20472307

[7] Xie Y Li HF Jiang B Li Y Kaminski HJ Kusner LL . Elevated plasma interleukin-17A in a subgroup of myasthenia gravis patients. Cytokine. (2016) 78:44–6. doi: 10.1016/j.cyto.2015.06.011 26618234

[8] Çebi M Durmus H Aysal F Özkan B Gül GE Çakar A . CD4+ T cells of myasthenia gravis patients are characterized by increased IL-21, IL-4, and IL-17A productions and higher presence of PD-1 and ICOS. Front Immunol. (2020) 11:809. doi: 10.3389/fimmu.2020.00809 PMC7248174 32508812

[9] Jia L Zhang L Liu M Ji H Wen Z Wang C . Mitochondrial control for healthy and autoimmune T cells. Cells. (2023) 12:1800. doi: 10.3390/cells12131800 37443834 PMC10340733

[10] Jiao Y Yan Z Yang A . Mitochondria in innate immunity signaling and its therapeutic implications in autoimmune diseases. Front Immunol. (2023) 14:1160035. doi: 10.3389/fimmu.2023.1160035 37122709 PMC10130412

[11] Shin B Benavides GA Geng J Koralov SB Hu H Darley-Usmar VM . Mitochondrial oxidative phosphorylation regulates the fate decision between pathogenic Th17 and regulatory T cells. Cell Rep. (2020) 30:1898–1909.e4. doi: 10.1016/j.celrep.2020.01.022 32049019 PMC9059282

[12] Sissler M González-Serrano LE Westhof E . Recent advances in mitochondrial aminoacyl-tRNA synthetases and disease. Trends Mol Med. (2017) 23:693–708. doi: 10.1016/j.molmed.2017.06.002 28716624

[13] Konovalova S Tyynismaa H . Mitochondrial aminoacyl-tRNA synthetases in human disease. Mol Genet Metab. (2013) 108:206–11. doi: 10.1016/j.ymgme.2013.01.010 23433712

[14] Kayvanpour E Wisdom M Lackner MK Sedaghat-Hamedani F Boeckel JN Müller M . VARS2 depletion leads to activation of the integrated stress response and disruptions in mitochondrial fatty acid oxidation. Int J Mol Sci. (2022) 23:7327. doi: 10.3390/ijms23137327 35806332 PMC9267100

[15] Bruni F Di Meo I Bellacchio E Webb BD McFarland R Chrzanowska-Lightowlers ZMA . Clinical, biochemical, and genetic features associated with VARS2-related mitochondrial disease. Hum Mutat. (2018) 39:563–78. doi: 10.1002/humu.23398 29314548 PMC5873438

[16] Nie A Sun B Fu Z Yu D . Roles of aminoacyl-tRNA synthetases in immune regulation and immune diseases. Cell Death Dis. (2019) 10:901. doi: 10.1038/s41419-019-2145-5 31780718 PMC6883034

[17] Ognjenović J Simonović M . Human aminoacyl-tRNA synthetases in diseases of the nervous system. RNA Biol. (2018) 15:623–34. doi: 10.1080/15476286.2017.1330245 PMC6103678 28534666

[18] Okuzono Y Miyakawa S Itou T Sagara M Iwata M Ishizuchi K . B-cell immune dysregulation with low soluble CD22 levels in refractory seronegative myasthenia gravis. Front Immunol. (2024) 15:1382320. doi: 10.3389/fimmu.2024.1382320 38711503 PMC11071663

[19] Leite MI Jacob S Viegas S Cossins J Clover L Morgan BP . IgG1 antibodies to acetylcholine receptors in ‘seronegative’ myasthenia gravis. Brain. (2008) 131:1940–52. doi: 10.1093/brain/awn092 18515870 PMC2442426

[20] Porcu E Sadler MC Lepik K Auwerx C Wood AR Weihs A . Differentially expressed genes reflect disease-induced rather than disease-causing changes in the transcriptome. Nat Commun. (2021) 12:5647. doi: 10.1038/s41467-021-25805-y 34561431 PMC8463674

[21] Nathan A Asgari S Ishigaki K Valencia C Amariuta T Luo Y . Single-cell eQTL models reveal dynamic T cell state dependence of disease loci. Nature. (2022) 606:120–28. doi: 10.1038/s41586-022-04713-1 35545678 PMC9842455

[22] Huda RH . Inflammation and autoimmune myasthenia gravis. Front Immunol. (2023) 14:1110499. doi: 10.3389/fimmu.2023.1110499 36793733 PMC9923104

[23] Lee GR . The balance of Th17 versus Treg cells in autoimmunity. Int J Mol Sci. (2018) 19:730. doi: 10.3390/ijms19030730 29510522 PMC5877591

[24] Wen Y Yang B Lu J Zhang J Yang H Li J . Imbalance of circulating CD4+CXCR5+FOXP3+ Tfr-like cells and CD4+CXCR5+FOXP3-2011; Tfh-like cells in myasthenia gravis. Clin Exp Med. (2016) 16:565–73. doi: 10.1016/j.neulet.2016.07.049 27473945

[25] Ashida S Ochi H Hamatani M Fujii C Kimura K Okada Y . Immune skew of circulating follicular helper T cells associates with myasthenia gravis severity. Neurol Neuroimmunol Neuroinflamm. (2021) 8:e945. doi: 10.1212/NXI.0000000000000945 33436376 PMC8105905

[26] Wu YW Luo JL Garden OA . Immunoregulatory cells in myasthenia gravis. Front Neurol. (2020) 11:593431. doi: 10.3389/fneur.2020.593431 33384654 PMC7769807

[27] Maiti A Kim JI . The alterations in and the role of the Th17/Treg balance in metabolic diseases. Front Immunol. (2021) 12:678355. doi: 10.3389/fimmu.2021.678355 34322117 PMC8311559

[28] Tyynismaa H . Disease models of mitochondrial aminoacyl-tRNA synthetase defects. J Inherit Metab Dis. (2023) 46:406–21. doi: 10.1002/jimd.12652 37410890

[29] Zhao R Luo S Zhao C . The role of innate immunity in myasthenia gravis. Autoimmun Rev. (2021) 20:102800. doi: 10.1016/j.autrev.2021.102800 33722749

[30] Dang EV Barbi J Yang HY Jinasena D Yu H Zheng Y . Control of TH17/Treg balance by hypoxia-inducible factor 1. Cell. (2011) 146:772–84. doi: 10.1016/j.cell.2011.07.033 21871655 PMC3387678

[31] Payet CA You A Fayet OM Hemery E Truffault F Bondet V . Central role of macrophages and nucleic acid release in myasthenia gravis thymus. Ann Neurol. (2023) 93:643–54. doi: 10.1002/ana.26590 36571580

[32] Verdier J Fayet OM Hemery E Truffault F Pinzón N Demeret S . Single-cell mass cytometry on peripheral cells in myasthenia gravis identifies dysregulation of innate immune cells. Front Immunol. (2023) 14:1083218. doi: 10.3389/fimmu.2023.1083218 36793723 PMC9922723

[33] Meraouna A Cizeron-Clairac G Le Panse R Bismuth J Truffault F Tallaksen C . The chemokine CXCL13 is a key molecule in autoimmune myasthenia gravis. Blood. (2006) 108:432–40. doi: 10.1182/blood-2005-06-2383 16543475 PMC1847364

[34] Shiao YM Lee CC Hsu YH Huang SF Lin CY Li LH . Ectopic and high CXCL13 chemokine expression in myasthenia gravis with thymic lymphoid hyperplasia. J Neuroimmunol. (2010) 221:101–6. doi: 10.1016/j.jneuroim.2010.02.013 20223524

[35] Võsa U Claringbould A Westra HJ Bonder MJ Deelen P Zeng B . Large-scale cis- and trans-eQTL analyses identify thousands of genetic loci and polygenic scores that regulate blood gene expression. Nat Genet. (2021) 53:1300–10. doi: 10.1038/s41588-021-00913-z 34475573 PMC8432599

[36] GTEx Consortium . The GTEx Consortium atlas of genetic regulatory effects across human tissues. Science. (2020) 369:1318–30. doi: 10.1126/science.aaz1776 32913098 PMC7737656

[37] Chia R Saez-Atienzar S Murphy N Chio A Blauwendraat C Roda RH . Identification of genetic risk loci and prioritization of genes and pathways for myasthenia gravis: a genome-wide association study. Proc Natl Acad Sci USA. (2022) 119(5):e2108672119. doi: 10.1073/pnas.2108672119 35074870 PMC8812681

[38] Kurki MI Karjalainen J Palta P Sipila TP Kristiansson K Donner KM . FinnGen provides genetic insights from a well-phenotyped isolated population. Nature. (2023) 613:508–18. doi: 10.1038/s41586-022-05473-8 36653562 PMC9849126

[39] Willer CJ Li Y Abecasis GR . METAL: fast and efficient meta-analysis of genomewide association scans. Bioinformatics. (2010) 26:2190–1. doi: 10.1093/bioinformatics/btq340 20616382 PMC2922887

[40] Zhu Z Zhang F Hu H Bakshi A Robinson MR Powell JE . Integration of summary data from GWAS and eQTL studies predicts complex trait gene targets. Nat Genet. (2016) 48:481–7. doi: 10.1038/ng.3538 27019110

[41] Chen J Xu F Ruan X Sun J Zhang Y Zhang H . Therapeutic targets for inflammatory bowel disease: proteome-wide Mendelian randomization and colocalization analyses. EBioMedicine. (2023) 89:104494. doi: 10.1016/j.ebiom.2023.104494 36857861 PMC9986512

[42] Wallace C . A more accurate method for colocalisation analysis allowing for multiple causal variants. PLoS Genet. (2021) 17:e1009440. doi: 10.1371/journal.pgen.1009440 34587156 PMC8504726

[43] Chen S Huang W Wan Q Tang Z Li X Zeng F . Investigation of the acute pathogenesis of spondyloarthritis/HLA-B27-associated anterior uveitis based on genome-wide association analysis and single-cell transcriptomics. J Transl Med. (2024) 22:271. doi: 10.1186/s12967-024-05077-y 38475831 PMC10936029

[44] Hao Y Stuart T Kowalski MH Choudhary S Hoffman P Hartman A . Dictionary learning for integrative, multimodal and scalable single-cell analysis. Nat Biotechnol. (2024) 42:293–304. doi: 10.1038/s41587-023-01767-y 37231261 PMC10928517

[45] Ianevski A Giri AK Aittokallio T . Fully-automated and ultra-fast cell-type identification using specific marker combinations from single-cell transcriptomic data. Nat Commun. (2022) 13. doi: 10.1038/s41467-022-28803-w 35273156 PMC8913782

[46] Lyu Z Kou Y Fu Y Xie Y Yang B Zhu H . Comparative transcriptomics revealed neurodevelopmental impairments and ferroptosis induced by extremely small iron oxide nanoparticles. Front Genet. (2024) 15:1402771. doi: 10.3389/fgene.2024.1402771 38826799 PMC11140123

[47] Lu S Lyu Z Wang Z Kou Y Liu C Li S . Lipin 1 deficiency causes adult-onset myasthenia with motor neuron dysfunction in humans and neuromuscular junction defects in zebrafish. Theranostics. (2021) 11:2788–805. doi: 10.7150/thno.53330 33456573 PMC7806489

[48] Zhu H Wang Z Wang W Lu Y He YW Tian J . Bacterial quorum-sensing signal DSF inhibits LPS-induced inflammations by suppressing toll-like receptor signaling and preventing lysosome-mediated apoptosis in zebrafish. Int J Mol Sci. (2022) 23:7110. doi: 10.3390/ijms23137110 35806111 PMC9266882

[49] Lyu Z Da Y Liu H Wang Z Zhu Y Tian J . Chsy1 deficiency reduces extracellular matrix productions and aggravates cartilage injury in osteoarthritis. Gene. (2022) 827:146466. doi: 10.1016/j.gene.2022.146466 35390446

